# Typical CT and MRI Features of Pancreatic Acinar Cell Carcinoma: Main teaching point: Typical imaging features of pancreatic acinar cell carcinoma are relatively large, with a well-defined margin, exophytic growth, and heterogeneous enhancement

**DOI:** 10.5334/jbsr.1854

**Published:** 2019-06-28

**Authors:** Kyoung A. Baek, Seung Soo Kim, Hyoung Nam Lee

**Affiliations:** 1 Department of Radiology, Soonchunhyang University College of Medicine, Cheonan Hospital, Cheonan-si, KR

**Keywords:** pancreatic neoplasms, acinar cell carcinoma, computed tomography, magnetic resonance imaging

## Case History

**Figure 1:**
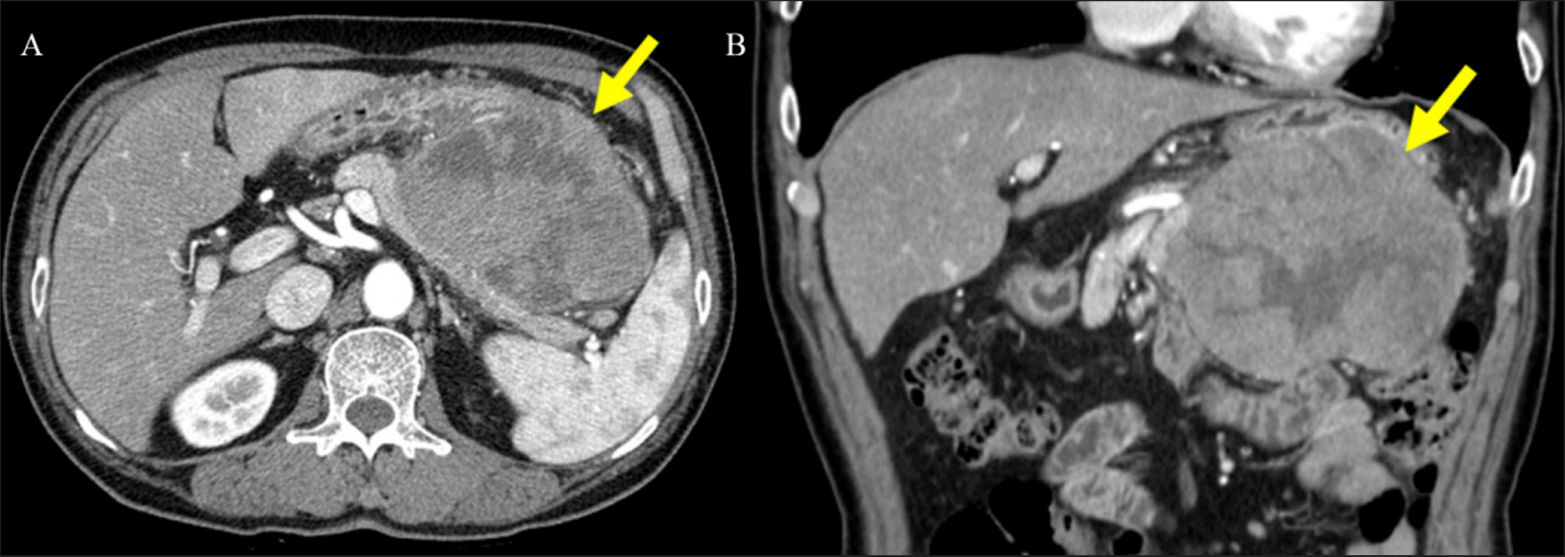


**Figure 2:**
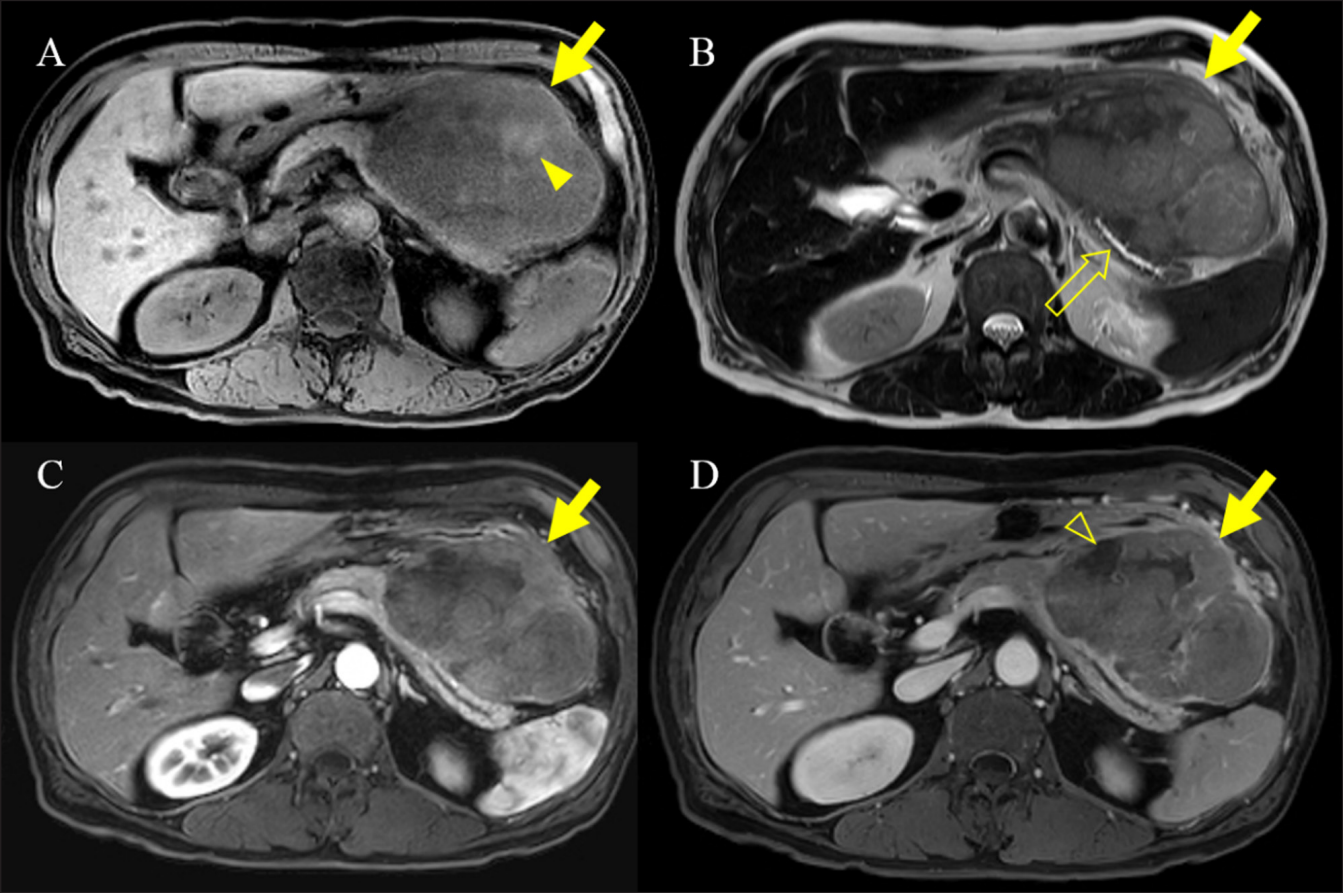


**Figure 3:**
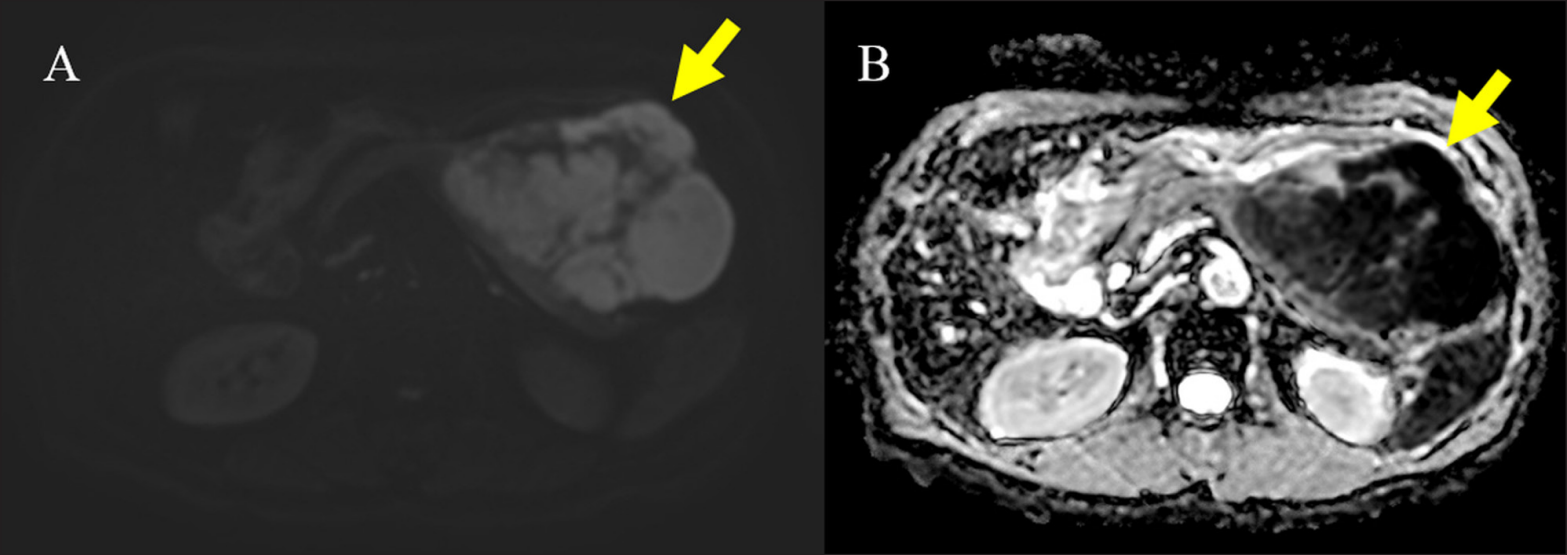


A 73-year-old man was admitted for evaluation of a pancreatic mass that was incidentally detected at a local clinic. He had a history of a 5 kg weight loss over a one-month period. The serum lipase was slightly elevated (103 IU/L), but amylase and tumor markers were within the normal range as follows: amylase, 79 IU/L; carbohydrate antigen (CA 19-9), 2.71 U/ml; and carcinoembryonic antigen (CEA), 1.75 ng/ml. Contrast-enhanced computed tomography (CT) (Figure 1) demonstrated a 12 cm, heterogeneous enhancing mass (arrow) in the pancreatic body and tail. Magnetic resonance imaging (MRI) (Figure 2) showed hemorrhage (arrowhead) and necrosis (open arrowhead) within the mass (arrow). The mass (arrow) revealed delayed enhancement on a dynamic gadolinium-enhanced image, and the pancreatic ductal dilatation (open arrow) was not severe compared with the mass size. On diffusion-weighted imaging (b = 800 s/mm^2^) and apparent diffusion coefficient map (Figure 3), the mass (arrow) showed diffusion restriction. The patient underwent distal pancreatectomy and was diagnosed with acinar cell carcinoma (ACC).

## Comment

ACC is a rare neoplasm of the pancreas (<1% of pancreatic neoplasms). ACC usually occurs in the fifth to seventh decades of life and arises in male patients more than in female patients [1]. This pancreatic tumor produces lipase, which occasionally causes polyarthritis and fat necrosis. Surgical resection is the treatment of choice for ACC. Although ACC is a malignant neoplasm, its prognosis is better than that of pancreas ductal adenocarcinoma (PDAC) [1].

Regarding their appearance on imaging, both ACCs and PDACs show hypovascularity compared with the pancreas parenchyma, but ACCs have the following characteristic features that are helpful in differentiating them from PDACs: relatively larger size (average, 6 cm), exophytic growth, a well-defined margin with enhanced capsule, lack of or relatively mild pancreatic duct dilatation or vascular encasement, internal necrosis, and cystic changes [1]. Solid pseudopapillary tumor (SPT) can show similar imaging findings to ACC, but SPT usually occurs in young women, in whom ACC rarely develops [1].
